# β-lapachone suppresses tumour progression by inhibiting epithelial-to-mesenchymal transition in NQO1-positive breast cancers

**DOI:** 10.1038/s41598-017-02937-0

**Published:** 2017-06-02

**Authors:** Yang Yang, Xianchun Zhou, Ming Xu, Junjie Piao, Yuan Zhang, Zhenhua Lin, Liyan Chen

**Affiliations:** 1 Department of Pathology & Cancer Research Center, Yanbian University Medical College, Yanji, 133002 China; 2 Department of Internal Medicine, Yanbian University Hospital, Yanji, 133000 China

## Abstract

NQO1 is a FAD-binding protein that can form homodimers and reduce quinones to hydroquinones, and a growing body of evidence currently suggests that NQO1 is dramatically elevated in solid cancers. Here, we demonstrated that NQO1 was elevated in breast cancer and that its expression level was positively correlated with invasion and reduced disease free survival (DFS) and overall survival (OS) rates. Next, we found that β-lapachone exerted significant anti-proliferation and anti-metastasis effects in breast cancer cell lines due to its effects on NQO1 expression. Moreover, we revealed that the anti-cancer effects of β-lapachone were mediated by the inactivation of the Akt/mTOR pathway. In conclusion, these results demonstrated that NQO1 could be a useful prognostic biomarker for patients with breast cancer, and its bioactivatable drug, β-lapachone represented a promising new development and an effective strategy for indicating the progression of NQO1-positive breast cancers.

## Introduction

Breast cancer is a common malignancy and a significant cause of death among females worldwide^1, 2^. Every year, breast cancer causes over 500,000 deaths. Recently, the advent of targeted therapies that include tyrosine kinase inhibitors (TKIs), such as lapatinib and anti-HER2 antibodies (such as trastuzumab), have considerably improved the overall survival and time-to-disease progression values in HER2+ breast cancer patients^3^. Although encouraging progress has been made in recent years due to the development of targeted therapy, the prognosis for breast cancer remains poor due to invasion and metastasis. The recurrence of breast cancer remains the critical clinical events associated with breast cancer. Therefore, extensive efforts are required to explore novel therapeutic targets to control the invasion and metastasis of breast cancer and to improve the quality of life among breast cancer patients.

In recent years, NAD(P)H:quinone oxidoreductase-1 (NQO1, EC 1.6.99.2) is a flavoprotein overexpressed up to 5- to 200-fold compared to normal adjacent tissue in various solid tumors, including cancers of the pancreas, lung, prostate and breast^4–6^, in terms of drug development. NQO1 is also an inducible phase II detoxifying 2-electron oxidoreductase that is capable of reducing most quinines and forming stable hydroquinones. Glutathione S-transferase then detoxifies hydroquinones and conjugates them with glutathione for secretion^7^. However, certain rare compounds, may undergo NQO1-mediated bioreduction for antitumour activity. Rather than detoxification, NQO1 converts specific quinones into highly cytotoxic species. Most antitumour quinones that depend on NQO1 are DNA alkylators, such as β-lapachone^8^ and mitomycin C^9^.

β-lapachone (β-lap) is a natural o-naphthoquinone compound that is obtained from the bark of the lapacho tree^10^. Its inner bark is often used as an analgesic, antiinflammatory, antineoplasic, antimicrobial and diuretic agent in the northeast of Brazil^11^. Interestingly, recent studies had proven that β-lap had a good antitumour effects on several carcinomas^12^, including hepatomas^13^, osteosarcomas^14^, breast cancers^15^, prostate cancers^16^ and human leukaemia^17^. Wu *et al*. also reported that β-lap effectively inhibited angiogenesis by suppressing tube formation and the invasion of HUVECs *in vitro*, and suppressed the growth and angiogenesis of human lung cancer xenografts in nude mice^18^. Additionally, numerous lines suggested that the mechanism for which β-lap had become a promising candidate for cancer therapy was dependent on the enzymatic activity of the two-electron oxidoreductase, NQO1^19, 20^. However, the expression level of NQO1 and the therapeutic potential of β-lap in breast cancer are unknown.

Thus, in the present study, we detected the expression level of NQO1 in breast cancers, and validated the therapeutic effects of β-lap in breast cancers cells. The results revealed that NQO1 expression was significantly up-regulated in breast cancers. Additionally, we demonstrated that β-lap inhibited the proliferation, invasion and migration of breast cancer cells and suppressed EMT by inactivating the Akt/mTOR pathway both *in vitro* and *in vivo*.

## Results

### NQO1 is overexpressed in breast cancers and correlated with invasion and metastasis

**Figure 1:**
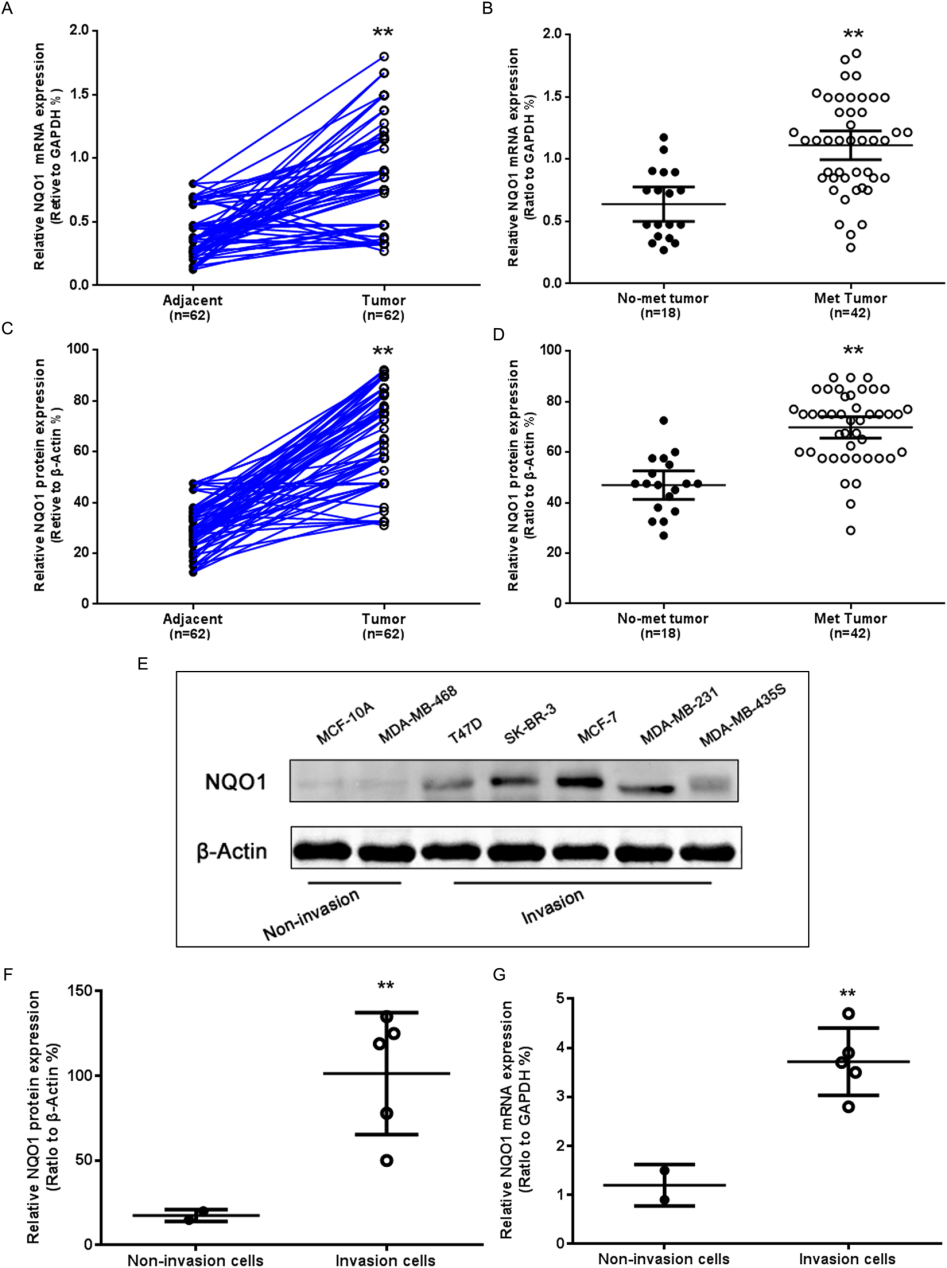
NQO1 was highly expressed in breast cancer. (**A**) The NQO1 mRNA expression levels in breast tumours and adjacent tissues were analysed via qRT-PCR. (**B**) Comparison of the NQO1 mRNA expression levels in the non-metastatic and metastatic breast cancer tissues. (**C**) Comparison of the NQO1 protein expression levels in the breast tumours and adjacent tissues. (**D**) Comparison of the NQO1 protein expression levels in the non-metastatic and metastatic breast cancer tissues. (**E**) The NQO1 protein expression levels in the breast cancer cell lines were analysed by western blot. (**F**) Comparison of the relative NQO1 protein expression levels in the invasive and non-invasive breast cancer cell lines. (**G**) Comparison of the relative NQO1 mRNA expression levels in invasive and non-invasive breast cancer cell lines. **P < 0.01 is based on the Student t test. All results are from three independent experiments. Error bars, SD.

To investigate whether NQO1 is highly expressed in breast cancers, both the mRNA and protein expression levels were detected by qRT-PCR and western blot in 62 samples of breast cancer tissues and matched normal adjacent non-tumour breast tissues. As compared with normal breast tissues, both the mRNA and protein expression levels of NQO1 were greater in the breast cancer specimens (Fig. 1A,C). Additionally, NQO1 was overexpressed in breast cancer patients with metastasis compared with patients without metastasis (Fig. 1B,D). As illustrated in Fig. 1E,F and G, the NQO1 protein and mRNA expression levels were higher in the invasive breast cancer cell lines than those in the non-invasive breast cancer cell lines. These data indicated that NQO1 might be relevant to the development and invasion of breast cancers.

**Figure 2:**
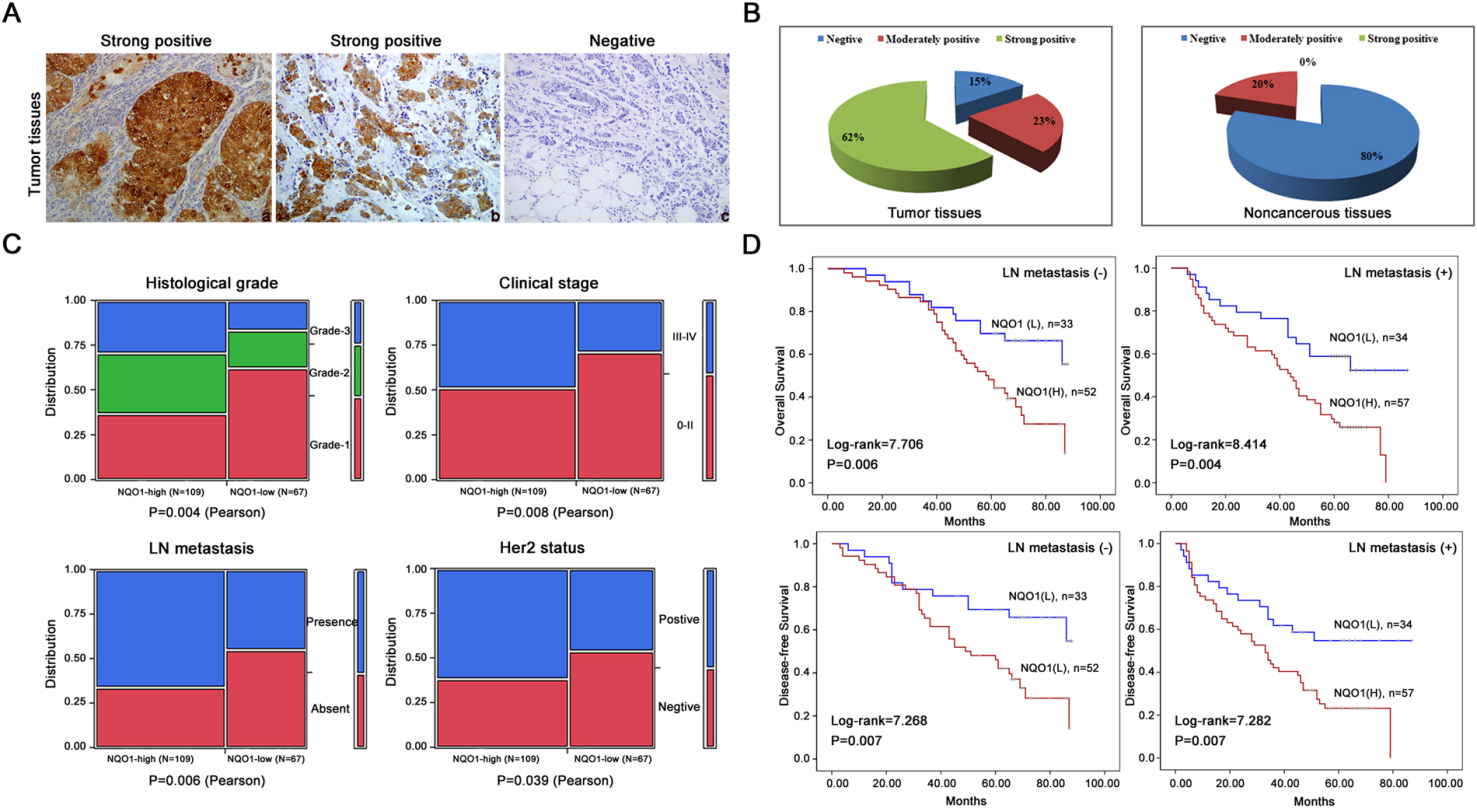
NQO1 over expression predicted poor prognosis of breast cancer. (**A**) NQO1 protein expressions in 221 cases of breast cancers were analyzed by IHC, and representative results are shown. (a) NQO1 staining was strongly positive in the breast cancer tissue. (b) NQO1 was strongly positive in the metastatic breast cancer cases. (c) NQO1 staining was negative in the non-metastatic breast cancer tissues. (**B**) The percentages of breast cancer tissues exhibiting negative, moderately positive and strongly positive of NQO1 expression in breast cancer tissues was calculated. (**C**) The relationships between NQO1 expression and the clinicopathologically significant aspects of breast cancer. (**D**) Kaplan-Meier survival curves for the breast cancer patients with and without lymph node metastasis. **P < 0.01 is based on the Student t test. All results are from three independent experiments. Error bars, SD.

IHC analyses were also performed to evaluate NQO1 expression in breast cancer patient samples (Fig. 2A). We observed that the positive rate of NQO1 expression was markedly higher in the breast cancer tissues than that in the adjacent non-tumour tissues (Fig. 2B and C). Additionally, we investigated the relationship between NQO1 expression and the clinicopathological parameters of the 221 cases of breast cancers, and significant correlations of NQO1 expression with tumour grade, clinical stage, Her2 status and lymph node metastasis (Fig. 2D). Kaplan-Meier analysis revealed that the patients with high NQO1 expression exhibited decreased DFS and OS compared to those with low NQO1 expression in both the LN metastasis (−) cases (*P* = 0.006 and *P* = 0.004, respectively) and the LN metastasis (+) cases (*P* = 0.007 and *P* = 0.007, respectively, Fig. 2E). These results collectively indicated a functional role of NQO1 in the aggressive behaviours of breast cancers, and NQO1 expression may predict longer survival and the lack of lymph vessel invasion in breast cancer patients.

### β-lapachone inhibited the proliferation of breast cancer cells in a dose- and time-dependent manner

**Figure 3:**
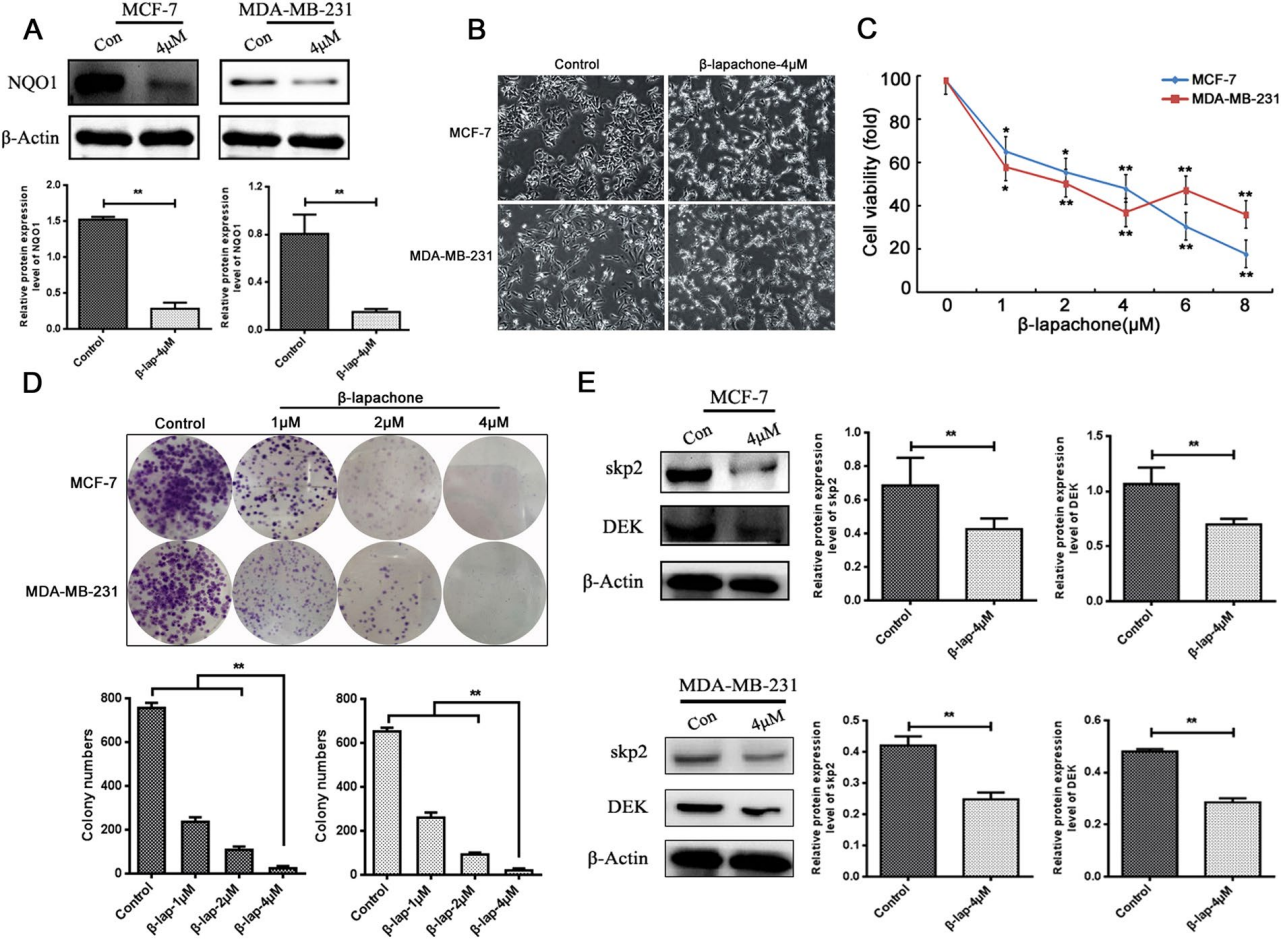
The effect of β-lapachone on breast cancer cell viability. (**A**) β-lap inhibited NQO1 in the MCF-7 and MDA-MB-231 cells. (**B**) The cell morphologies of the MCF-7 and MDA-MB-231 cells that were treated with the indicated concentrations of β-lap were determined by interference light microscopy (magnified x 200). (**C**) The viabilities of the MCF-7 and MDA-MB-231 cells that were treated with β-lap were measured via MTT assay. (**D**) The colony formation capacities were detected among the MCF-7 and MDA-MB-231 cells following β-lap treatment. (**E**) Skp2 and DEK expressions were detected among the MCF-7 and MDA-MB-231 cells following β-lap treatment by western blot. β-Actin was used as a loading control. **P < 0.01 is based on the Student t test, One-Way Anova. All results are from three independent experiments. Error bars, SD.

β-lap is a novel radiosensitizer with potent antitumour efficacy that selectively kills solid cancers that over-expressed NQO1^21^. To investigate the antitumour effects of β-lap in breast cancer cells, MCF-7 and MDA-MB-231 breast cancer cell lines, which both over-expressed NQO1 (Fig. 1E), were selected. First, we confirmed the effects of NQO1 inhibition, and the results revealed that β-lap significantly inhibited NQO1 protein expression in over-expressed NQO1 breast cancer cells (Fig. 3A). As illustrated in Fig. 3B, β-lap improved the morphological appearance of dying cells in a dose-dependent manner, and cell viability was decreased in the β-lap-treated cells (Fig. 3C). Similarly, the colony formation capacities were markedly inhibited in both the MCF-7 and MDA-MB-231 cells following β-lap treatment (Fig. 3D).

Skp2 and DEK played important roles in oncogenesis and regulate the expressions of various proteins that are involved in cell cycle arrest^22, 23^. Gao *et al*. suggested that arsenic trioxide inhibited cell growth and invasion via down-regulation of Skp2 in pancreatic cancer cells^24^. Yu *et al*. reported that DEK promoted cell proliferation through the regulation of cell cycle-related genes in HCC cells^23^. Thus, to further investigate the molecular mechanism by which of β-lap inhibited breast cancer cell proliferation, DEK and Skp2 expression were detected in MCF-7 and MDA-MB-231 cells following β-lap treatment. The results revealed that β-lap down-regulated Skp2 and DEK expression in MCF-7 and MDA-MB-231 breast cancer cell lines (Fig. 3E and F). These results indicated that β-lap inhibited breast cancer cell proliferation by down-regulating Skp2 and DEK expression.

### β-lapachone inhibited migration and invasion by suppressing EMT progression in NQO1-positive breast cancer cells

**Figure 4:**
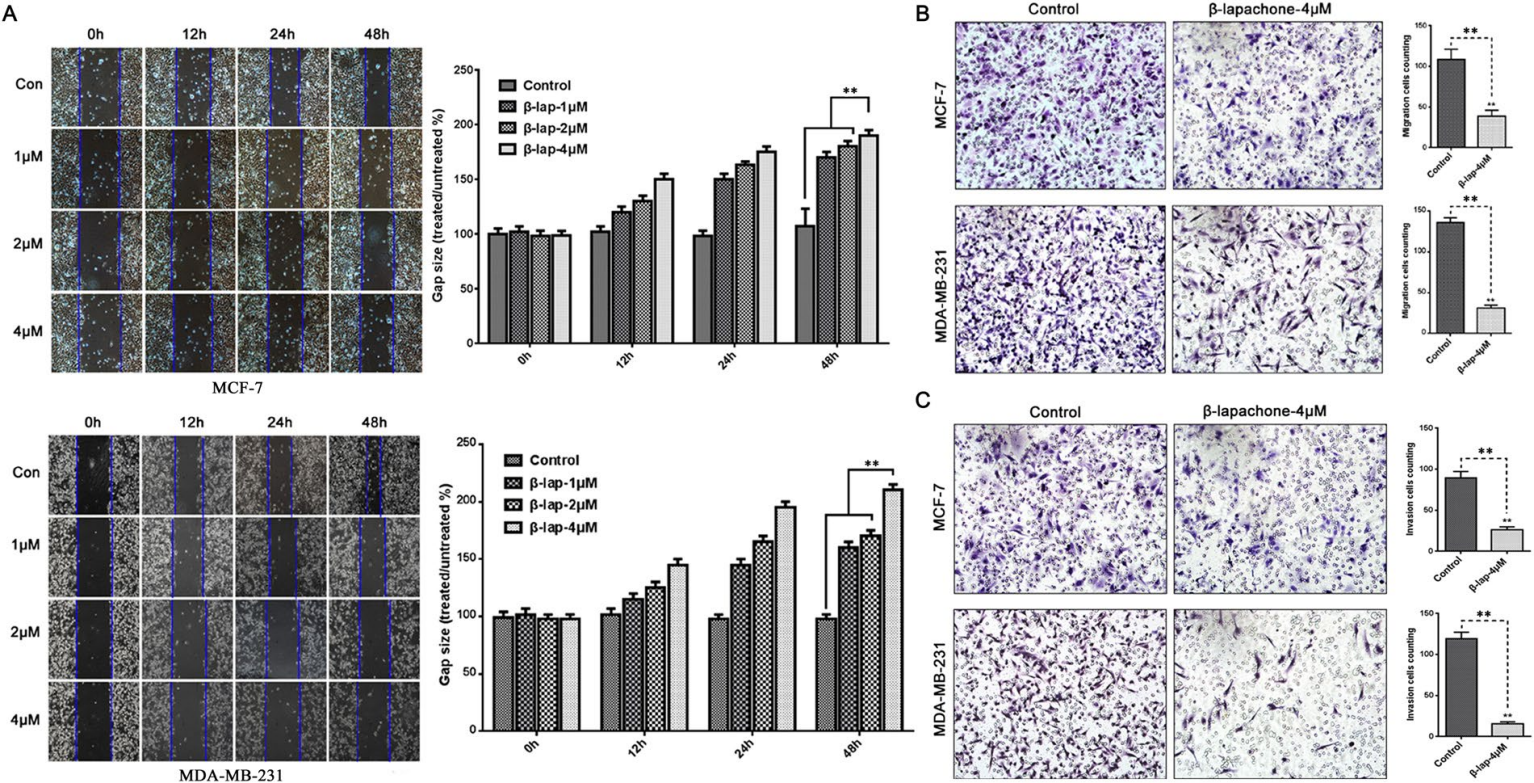
β-lapachone suppressed the migratory and invasive capacities of the breast cancer cells. (**A**) MCF-7 and MDA-MB-231 cells were treated with DMSO (control), 1 μM, 2 μM, 4 μM β-lap for 12 h, 24 h, or 48 h after scratching. The uncovered areas in the wound healing assays were quantified according to the percentage of the original wound area. (**B**) MCF-7 and MDA-MB-231 cells were treated with 4 μM β-lap for 48 h in the transwell migration assays. The numbers of invading cells were calculated in three different fields (200 × ). (**C**) MCF-7 and MDA-MB-231 cells were treated with 4 μM β-lap for 48 h in the Boyden chamber assay. The numbers of invading cells were calculated in three different fields (200×). **P < 0.01 is based on One-Way Anova. All results are from three independent experiments. Error bars, SD.

**Figure 5:**
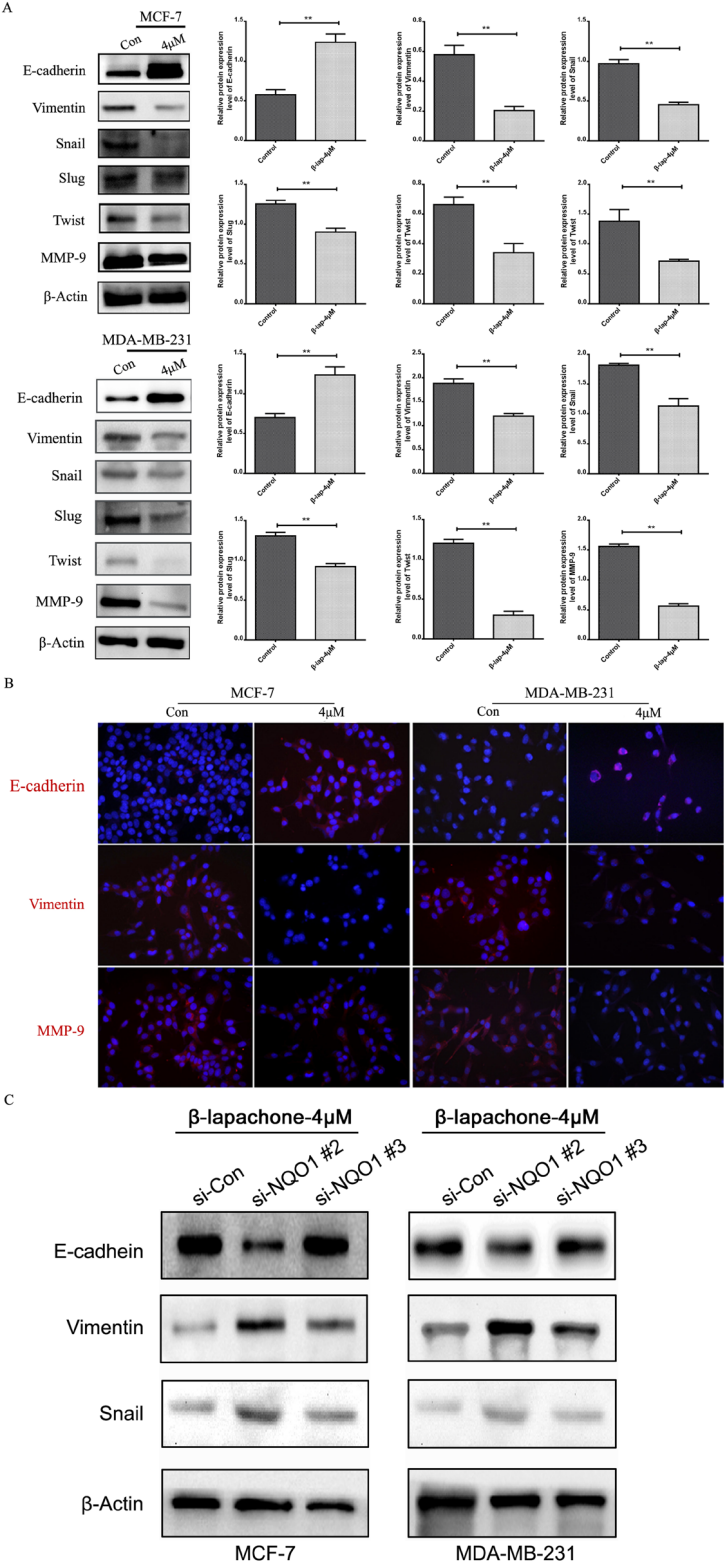
β-lapachone suppressed EMT progression in breast cancer cells *in vitro*. (**A**) The changes in the protein expression levels of the epithelial marker E-cadherin and the mesenchymal markers Vimentin, Snail, Slug, Twist, and MMP-9 in MCF-7 and MDA-MB-231 cells treated with DMSO or 4 μM β-lap for 48 h. (**B**) IF staining for the E-cadherin, Vimentin, and MMP-9 proteins in the two groups of MCF-7 and MDA-MB-231 cells treated with 4 μM β-lap. (**C**) The changes in the protein expression levels of the epithelial marker E-cadherin and the mesenchymal markers Vimentin, snail in si-NQO1-transfected MCF-7 and MDA-MB-231 cells treated with 4 μM β-lapachone for 48 h. **P < 0.01 is based on the Student t test. All results are from three independent experiments. Error bars, SD.

As is well known, migration and invasion remain the critical clinical events related to cancers. Thus, to evaluate the effects of β-lap on in terms of the suppression of migration and invasion, we used the wound healing, migration and transwell assays. As illustrated in Fig. 4A–C, the results indicated that β-lap suppressed the migratory and invasive behaviors of breast cancer cells. EMT plays important roles in cancer invasion, migration and metastasis, and in the present study, we found that β-lap significantly reduced cell migration and invasion. Therefore, we hypothesized that β-lap reduced cell migration and invasion by suppressing EMT progression. Therefore, the expression levels of EMT markers were detected with western blot in MCF-7 and MDA-MB-231 breast cancer cells following β-lap treatment. Epithelial markers E-cadherin was significantly increased, while the mesenchymal markers Vimentin, Slug and MMP-9 were decreased, and the transcription factors Snail and Twist were also decreased (Fig. 5A). Consistently, IF analysis also revealed that β-lap significantly increased E-cadherin expression and decreased the expressions of Vimentin and MMP-9 in the MCF-7 and MDA-MB-231 breast cancer cell lines (Fig. 5B). These results suggested that β-lap suppressed EMT in breast cancer cells *in vitro*.

**Figure 6:**
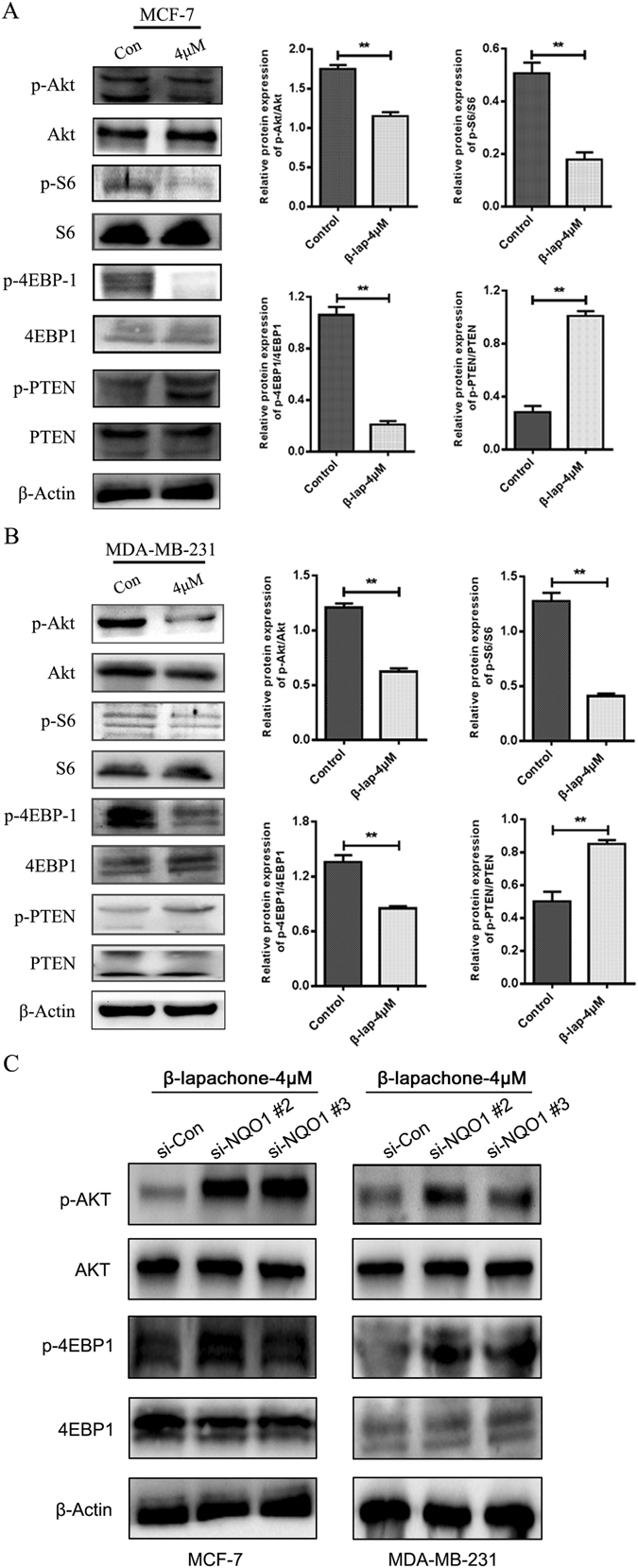
β-lapachone inhibited the Akt/mTOR signalling pathway in breast cancer cells *in vitro*. (**A**) Western blots for Akt/mTOR pathway inhibition in MCF-7 and MDA-MB-231 cells treated with DMSO or 4 μM β-lap for 48 h. (**B**) Western blots for Akt/mTOR pathway inhibition in si-NQO1-transfected MCF-7 and MDA-MB-231 cells treated with 4 μM β-lapachone for 48 h. **P < 0.01 is based on the Student t test. All results are from three independent experiments. Error bars, SD.

Furthermore, we investigated the signaling mechanisms that were involved in the β-lapachone-mediated EMT process and the proliferation of inhibition by determining the effects of β-lap on the activities of Akt, S6, 4EBP-1 and PTEN. β-lap significantly down-regulated the expressions of the activated forms of Akt and mTOR as indicated by the decreased levels of phosphorylation of the Akt, S6, and 4EBP1 proteins and the increased levels of phosphorylation of PTEN in the absence of alterations of the total levels (Fig. 6A, *P* < 0.05). These results suggested that β-lap inhibited EMT and proliferation at least partially by up-regulating the phosphoinositide 3-kinase (PI3K)/Akt/mTOR signalling pathways.

To identify the function of NQO1 in the global change induced by β-lap treatment, we knocked down NQO1 in high-invasive breast cancer cell lines MCF-7 and MDA-MB-231 (Supplemental Fig. 1). Then we treated breast cancer cells with β-lap for 48 h. As illustrated in Supplemental Fig. 2, NQO1 knockdown significantly protected breast cancer cells from β-lapachone-induced cell death in MCF-7 and MDA-MB-231 cells, compared with control group. In addition, we investigated the role of NQO1 in breast cancer cell motility inhibited by β-lap using wound-healing assay. The results showed that after NQO1 knocked down, the inhibition on the migration of MCF-7 and MDA-MB-231 cells by β-lap were significantly reduced (Supplemental Fig. 3). Moreover, the inhibition of β-lap on EMT progress and AKT pathway were also attenuated with the silencing of NQO1 (Figs 5B and 6B). These data indicates that β-lap exerted the anti-tumour activity in breast cancer cells in a NQO1-dependent manner.

### β-lapachone inhibited tumour growth and invasion *in vivo*

**Figure 7:**
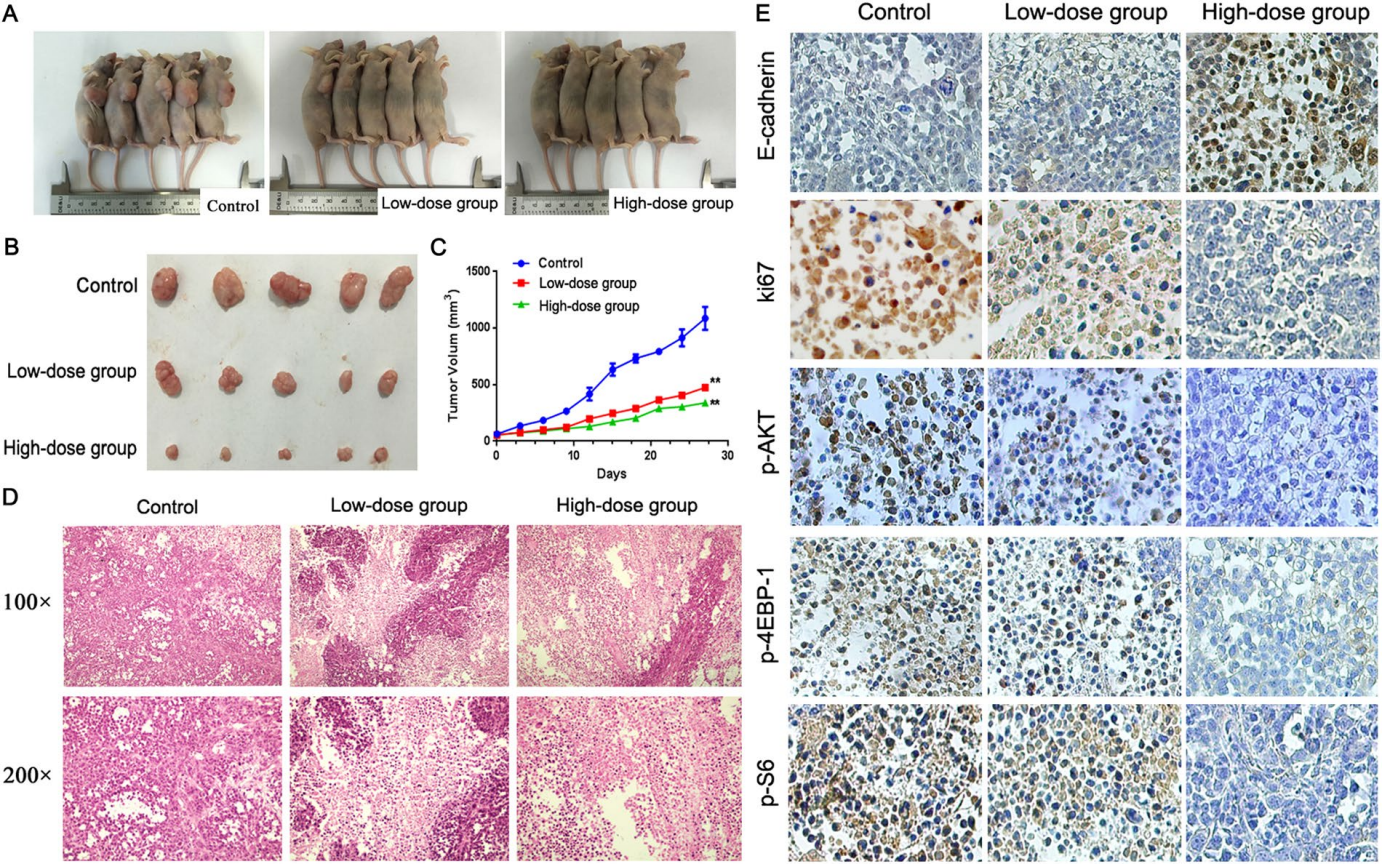
β-lapachone inhibitors effectively attenuate tumour growth and the proliferation of breast cancer cells in a mouse xenograft model. (**A**) The nude mice 27 days after injections and the growth curves of tumours. (**B**) and (**C**). Tumour volumes according to treatment group. (**D**) HE staining of the necrosis foci and lymphocyte infiltration of the tumours are noted (200 × ). (**E**) The expression levels of E-cadherin, Ki-67, p-Akt, p-S6, and p-4EBP1 in the tumour tissues were assayed (200×). **P < 0.01 is based on the Student t test, GLM-Repeated measurment. All results are from three independent experiments. Error bars, SD.

We determined the effects of β-lap on tumour growth using an established xenograft model generated by the subcutaneous dorsal implantation of MDA-MB-231 cells into nude mice. As illustrated in Fig. 7A–C, four weeks of treatment with β-lap reduced tumour growth compared with the control and resulted in a lower mean MDA-MB-231 tumour volume (*P* < 0.01). To further investigate the effects of these drugs treatments *in vivo*, tumours from 3 mice in each group were analyzed via H&E and immunohistochemical (IHC) staining. Interestingly, the H&E staining revealed increased necrosis in the low-dose β-lap-treated tumour group compared with the control group, and the level of necrosis was particularly high among the high-dose β-lap-treated tumour group (Fig. 7D). The IHC staining revealed increased expression of E-cadherin, and decreased expression of Ki-67, Vimentin, snail, phosphorylation of Akt, S6, and 4EBP1 in the high-dose β-lap-treated tumour group compared with the other groups (Fig. 7E, Supplemental Fig. 4).

Furthermore, we observed the effect of β-lap on breast cancer cell invasion by chorioallantoic membrane (CAM) invasion assay using hematoxylin and eosin (H&E) staining. Cells were cultured on top of the chick CAM. After cultured for 3 days, MCF-7 and MDA-MB-231 cells spread rapidly across the CAM surface and infiltrated the underlying stroma (Supplemental Fig. 5). The invasion was dramatically inhibited by β-lap treated, which indicating that β-lap inhibited the invasion of breast cancer cells *in vivo*.

## Discussion

For years, We have focused on exploiting elevations of the NQO1 levels in numerous solid tumors, particularly in non-small cell lung cancer (NSCLC), gastric cancer, pancreatic cancer, and breast cancer^25, 26^, for drug development. Increases or decreases in NQO1 protein levels in cancer cells could be dependent on the following different potential cooperative mechanisms: the corresponding genomic imbalances of the NQO1 locus, which is located on chromosomal band 16q22; the activation of the Nrf2 pathway; and/or the presence of NQO1 polymorphisms^27, 28^. In this study, we revealed that the overexpression of NQO1 was significantly correlated with metastasis in breast cancer specimens. Our findings further highlighted that the overexpression of NQO1 was associated with a decrease in the overall survival of breast cancer patients. These empirical findings suggest that breast cancer patients with high NQO1 levels in tissue samples have a greater chance of metastasis and significantly shorter overall and disease-free survivals.

β-lap is a well-known bioactivatable drug of NQO1, which catalyses the oxidation of NADPH to NAD^+^. Many researchers considered β-lap as a candidate anti-cancer agent. Kung *et al*. reported that β-lap inhibited the survival/proliferation of lung cancer cells via the PI3K/Akt/ERK pathway^21^. Jeon *et al*. also found that β-lap inhibited cell proliferation and induced apoptosis by targeting Sp1 and apoptosis associated proteins in non-small cell lung cancer^29^. Consistent with these results, we found that β-lap exhibited strong dose-dependent inhibitory effects on MCF-7 and MDA-MB-231 cell proliferation (*P* < 0.05). Additionally, we found that DEK and Skp2 regulation were involved in the mechanisms of these anti-cancer effects. However, further study is needed to explore the exact mechanisms of the regulations of DEK and Skp2 by β-lap.

EMT is believed to be a critical step in the progression of neoplasms because it confers migratory and invasive properties^30^. Although the roles of β-lap in tumour cell apoptosis abilities are well understood, the regulation of tumour cell migration and invasion activities by β-lap, including its role in EMT, remain elucidated. Kim *et al*. suggested that β-lap inhibited the progression and metastasis of hepatoma cells by inhibiting the invasion abilities of the cells via the up-regulation of the expressions of the Egr-1, TSP-1, and E-cadherin^31^. Moreover, Kung *et al*. first demonstrated that low concentrations of β-lap can increase the proliferation of cells that include keratinocytes, fibroblasts, and endothelial cells and the migration of fibroblasts and endothelial cells and that β-lap may have potential therapeutic uses in wound healing^32^. In this study, we demonstrated that β-lap up-regulated epithelial marker E-cadherin, and down-regulated Vimentin, twist, Slug, Snail, and MMP-9, which indicated that β-lap suppressed EMT progression in breast cancer cells. To further identify the role of NQO1 in the global changes induced by β-lap treatment, we knocked down NQO1 in high-invasive breast cancer cell lines MCF-7 and MDA-MB-231, then treated cells with β-lap. The data showed after NQO1 knocked down, the inhibition on the migration and EMT progress of breast cancer cells by β-lap were significantly reduced. These results suggested that β-lap exerted the EMT progress of breast cancer cells in a NQO1-dependent manner.

Previous experiments have demonstrated that β-lap significantly inhibited cellular proliferation and induced apoptosis in human gastric carcinoma AGS cells via the activation of the PI3K/Akt pathway and thus providing a mechanism for the role of β-lap in cell survival^33^. Our results indicated that β-lap decreased the levels of the phosphorylations of the Akt, S6, and 4EBP1 proteins (*P* < 0.05) and increased the levels of phosphorylation of PTEN (*P* < 0.05), but had no significant effect on the total levels of these proteins (*P* > 0.05). These results suggested that the Akt/mTOR pathway might participate in β-lapachone-mediated EMT suppression.

**Figure 8:**
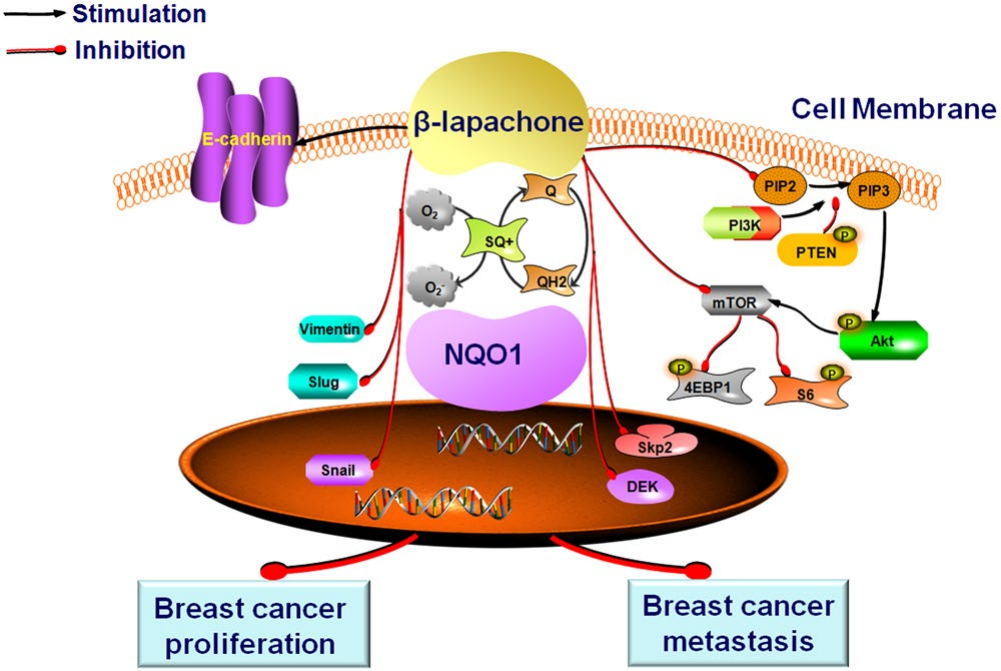
The potential role of β-lapachone in breast cancer and a diagrammatic sketch.

In summary, NQO1 overexpression was linked with poor clinical outcome and was implicated in breast cancer progression. As a NQO1 activator, β-lap exhibited a novel anti-cancer activity in NQO1-postive breast cancer cells. Additionally, β-lap inhibited breast cancer cell proliferation, invasion and migration. EMT progression was also suppressed by β-lap, and these anti-cancer effects may be due to Akt/mTOR pathway inactivation (Fig. 8). β-lap could represent as a promising new agent for the development of novel and effective strategies for preventing the progression of NQO1-postive breast cancer.

## Materials and Methods

### Ethics statement

This research complied with the Helsinki Declaration and was approved by the Human Ethics Committee and the Research Ethics Committee of Yanbian University Medical College. Patients were informed that the resected specimens were stored by the hospital and potentially used for scientific research, and that their privacy would be maintained. Follow-up survival data were collected retrospectively through medical record analyses.

### Clinical samples

62 fresh breast cancers paired with adjacent non-tumor tissues were snapfrozen in liquid nitrogen and stored at −80 °C until use. The histopathology of each specimen was reviewed on the hematoxylin and eosin-stained tissue section to confirm diagnosis and tumor content at least 70% of tumor cells in the tissue sample. The study of 176 paraffin embedded breast cancer samples, as well as 45 ductal carcinoma *in situ* (DCIS) samples, 22 hyperplasis and 52 adjacent non-tumor tissues were also conducted. These samples were selected randomly from patients who underwent surgery between 2002 and 2009, with strict follow-up for survival status. Clinicopathological classification and staging were determined according to the American Joint Committee on Cancer (AJCC) criteria.

### Cell culture

Human breast carcinoma cell lines (MCF-7, MDA-MB-231, MDA-MB-453, MDA-MB-468, SK-BR-3, T47D) were provided by Cancer Research Center of Yanbian University. The cells were cultured in Dulbecco’s modified Eagle’s medium (DMEM) or RPMI 1640 medium with 10% fetal bovine serum (FBS, Gibco) at a temperature of 37 °C under 5% CO_2_.

### Immunohistochemistry (IHC) of human breast cancer tissues

Human breast cancer specimens were obtained from 176 patients who underwent breast cancer surgeryat the Cancer Hospital of Shantou University Medical College, China between 2010 and 2011. Written informed consent was obtained from each patient, and the study was approved by the Hospital Research Ethics Committee.

Serial formalin-fixed and paraffin-embedded tissues were sectioned at a 4 μm thickness, deparaffinized, and rehydrated in gradients of high percentage ethanol to distilled water. For quenching endogenous peroxidase activity, sections were immersed in 3% hydrogen peroxide for 15 min at room temperature. Antigen retrieval involved boiling in 10 mM sodium citrate buffer (pH 6) for 3 min in a pressure cooker, followed by cooling to room temperature. Sections were then incubated with the primary antibody at 4 °C overnight, washed three times in PBS for 5 min, and incubated with horseradish peroxidaseconjugated goat anti-mouse/rabbit IgG antibody (ZSGBBio, Beijing, China) at room temperature for 30 min, followed by 3,3′-diaminobenzidine tetra-hydrochloride (DAB) staining. Sections were lightly counterstained with hematoxylin.

Two pathologists (Lin Z & Liu S) who did not possess knowledge of the clinical data examined and scored all tissue specimens. In case of discrepancies, a final score was established by reassessment by both pathologists on a double-headed microscope. Briefly, the IHC staining for NQO1 was semi-quantitatively scored as ‘−’ (negative) (no or less than 5% positive cells), ‘+’ (5–25% positive cells), ‘++’ (26–50% positive cells) and ‘+++’ (more than 50% positive cells). The cytoplasmic expression pattern was considered as positive staining. Tissue sections scored as ‘++’ and ‘+++’ were considered as strong positives (high level expression) of NQO1 protein.

### Quantitative real-time PCR (qRT-PCR)

As described previously^34^, total RNA samples from eight of primary tumor materials were extracted using Trizol reagent (Invitrogen, Carlsbad, CA, USA) according to the manufacturer’s instructions. The extracted RNA was pretreated with RNase-free DNase, and 2 µg RNA from each sample was used for cDNA synthesis primed with random hexamers. For the PCR amplification of NQO1 cDNA, an initial amplification step using NQO1 specific primers was performed with denaturation at 95 °C for 15 min, followed by 38 denaturation cycles at 95 °C for 30 s, primer annealing at 60 °C for 30 s, and a primer extension phase at 72 °C for 30 s. Upon the completion of the cycling steps, a final extension step at 72 °C for 7 min was conducted before the reaction mixture was stored at 4 °C. Real-time PCR was then employed to determine the fold increase of NQO1 mRNA in each of the primary breast tumors relative to the paired adjacent non-tumor tissue taken from the same patient. Double-stranded DNA specific expression was tested by the comparative Ct method using 2-∆∆Ct. Primer sets for specific genes are showed in Table 1. Expression data were normalized to the geometric mean of GAPDH to control the variability in expression levels. All experiments were performed in triplicate.

**Table 1:** List of primers used in this study.

Gene	Primer	Sequence
Primers for NQO1	forward	5′-GGCAGA AGAGCACTGATCGTA-3′
	reverse	5′-TGATGGGATTGA AGTTCATGGC-3′
Primers for GAPDH	forward	5′-CATCACCATCTTCCAGGAGCG-3′
	reverse	5′-TGACCTTGCCCACAGCCTTG-3′

### Western blot analysis

For western blot analysis, cells were harvested and lysed in lysis buffer. A total of 30 μg of protein lysates were separated by SDS-PAGE and transferred onto a PVDF membrane. Primary antibodies were diluted according to the company’s recommendation. Proteins were visualized using the enhanced chemiluminescence detection system.

### MTT assay

Cell viability was measured using the MTT [3-(4,5-dimethylthiazol-2-yl) -2,5-diphenyl tetrazolium] dye reduction method. In brief, cells were cultured in each of the wells of 96-well plates with varying concentrations of β-lap for 48 h. After addition of an MTT solution (0.5 mg/ml), the plates were incubated for 4 h, the media was removed, and the formazan crystals were then solubilized in dimethyl sulfoxide (DMSO). The formazan crystals in the cells were dissolved in DMSO, and the absorbance at 570 nm was then measured using a microplate reader (Bio-Tek Instruments, USA).

### Colony formation assay

Cells in the log growth phase were seeded at a density of 1 × 10^3^ cells per well in 6-well plates and left to attach overnight prior to treatment. Subsequently, the cells were treated with different concentrations of β-lap and compared with the DMSO-treated controls. After 14 days, the plates were stained with crystal violet, and the colonies containing more than 450 cells were counted using an Olympus phase-contrast microscope.

### Wound healing assay

The wound-healing assay was performed as described previously for migration analysis^35^. The cells were seeded in a 6-well plate and cultured for 24 h to form confluent monolayers. A wound was created by dragging a pipette tip through the monolayer, and the plates were washed using pre-warmed PBS to remove the cellular bdebris. Cell migration was monitored at 0 h and 48 h, and images were captured at each time point using a digital camera attached to an inverted phase contrast microscope.

### Invasion and migration assays

Twenty-four-well transwells which containing an 8-mm pore size poly-carbonate membrane with or without a Matrigel-coated membrane matrix were used to test the invasion or migration of the breast cancer cells. The cells (2 × 10^5^/ml) were resuspended in 200 μl of serumfree medium. The cells were then plated on the top side of the polycarbonate Transwell filter (without Matrigel for the Transwell assay) or plated on the top side of the polycarbonate Transwell filter coated with Matrigel (for the Transwell matrix penetration assay) in the upper chamber of a BioCoat Invasion Chambers (BD) and incubated at 37 °C for 48 h, followed by the removal of cells inside the upper chamber with cotton swabs. The migrated and invaded cells on the lower membrane surface were fixed in 4% formaldehyde and then stained with 0.1% crystal violet for 5 min. Five fields of cells were counted randomly in each well under a C microscope at 200x magnification.

### Immunofluorescence (IF) staining

IF staining was used to detect the sub-cellular localization of NQO1 protein in breast cancer cells. All steps were performed at RT. Breast cancer cells were grown on coverslips to 70–80% confluence, then fixed with 4% paraformaldehyde for 10 min and permeabilized with 0.5% TritonX-100 for 10 min after 24 h. After blocking with 3% Albumin Bovine V (A8020, Solarbio, Beijing, China) for 1 h, the slides were quickly and gently washed with PBS. The cells were then incubated with the E-cadherin, Vimentin antibody at 4 °C overnight, and followed by incubation with Alexa Fluor® 568 goat anti-mouse IgG (H + L) (A11004, 1:1000, Invitrogen, Carlsbad, CA, USA) for 1 h. After washing with PBS, cells were counterstained with 49–6-diamidino-2-phenylindole (DAPI) (C1006, Beyotime, Shanghai, China) and the coverslips were mounted with Antifade Mounting Medium (P0126, Beyotime)^27^. Finally, the IF signals were visualized under a Leica SP5II CLSM microscope (Heidelberg, Germany) with filters for the corresponding fluorescent stains.

### Transfection

We purchased two different NQO1 siRNA, including si-NQO1 #1, si-NQO1 #2 and si-NQO1 #3, from RIBOBIO (China). According to the KD effect, si-NQO1 #2 was used in this study. The sequence of si-NQO1 #2 was 5′-GGACGTCCTTCAACTATGC-3′, si-NQO1 #3 was CAACTGACATATAGCATTG. Additionally, control siRNA (si-control) was also used in this study. Cells were transfected with 30 nM siRNA using Lipofectamine 3000 (Invitrogen) according to the manufacturer’s instructions. For the KD effect of si-NQO1 #1, we showed the some results in Supplemental Fig. 3.

### Human tumour xenografts in severe combined immunodeficient (BALB-C/nude) mice

To test the effects on breast cancer *in vivo*, a xenograft model of human breast cancer was established. Four-week old female BALB-C/nude mice were purchased from Vital River Laboratory Animal Technology Co. Ltd., (Beijing, China). After 1 week of acclimatization, the BALB-C/nude mice were subcutaneously injected in the right flank with 5 × 10^6^ MDA-MB-231 breast cancer cells that were resuspended in 50 μl DMEM medium. The treatment was initiated when the subcutaneous tumors reached an average size of 150 to 200 mm^3^. Mice were randomized into three groups, which treated with normal saline, low-dose β-lap (12.5 mg/kg/day) and high-dose β-lap (25 mg/kg/day). Tumour diameters were measured every 2 days with callipers, and the tumour volumes were calculated (length × width × height × 0.5). All experiments were performed in keeping with the procedures and protocols of the Animal Ethics Committee of Yanbian University.

### Chick chorioallantoic membrane assays (CAM assay)

Fertilized chicken eggs were incubated in incubator at 37.8 °C with 60–65% humidity. Ethics approval was obtained by the University of Yanbian Animal Ethics Committee. MCF-7 and MDA-MB-231 breast cancer cells (control, β-lap-4 μm) *in vivo* were assessed using 11-day-old chick embryos in which an artificial air sac was created under aseptic conditions^36^. A total of 1 × 10^6^ cells were inoculated atop the chick chorioallantoic membrane (CAM) and the window was resealed with adhesive tape and eggs were returned to the incubator until day 14 of chick embryo development. The CAM was removed at the surrounding CAM were harvested from each embryo and fixed with 4% paraformaldehyde for 24 h and embedded in paraffin. Serial sections (4 μm) were stained with H&E.

### Statistical analysis

Each experiment was performed in triplicate. All data are presented as the mean ± SD. Statistical analysis was performed using the SPSS 17.0 statistical package (SPSS, Inc., Chicago, IL, USA), and comparisons between groups were conducted using One Way ANOVA. Survival rates were calculated using the Kaplan-Meier method, and differences in survival curves were analyzed by log-rank tests. Differences were considered statistically significant at *P* < 0.05. Tumor free survival was calculated with log-rank (Mantel-Cox) test using GraphPad Prism 5 (GraphPad Software, Inc., San Diego, CA, USA).

## Electronic supplementary material

Supplementary Information
