# Supplementary material for: β-lapachone suppresses tumour progression by inhibiting epithelial-to-mesenchymal transition in NQO1-positive breast cancers
Source: Sci Rep. 2017 Jun 2;7:2681. doi: 10.1038/s41598-017-02937-0 (PMC5457413; doi:10.1038/s41598-017-02937-0)
Supplement: Supplementary file 1 — Supplementary Information [file 41598_2017_2937_MOESM1_ESM.pdf]

Supplementary Information

β-lapachone suppresses tumour progression by inhibiting epithelial-to-mesenchymal transition in NQO1-positive breast cancers

Yang Yang[1]*, Xianchun Zhou[2]*, Ming Xu[1], Junjie Piao[1,2], Yuan Zhang[1], Zhenhua Lin[1#], Liyan Chen[1#]

1 Department of Pathology & Cancer Research Center, Yanbian University Medical College, Yanji 133002, China

2 Department of Internal Medicine, Yanbian University Hospital, Yanji 133000, China

*These authors contributed equally to this work.

Correspondence to:

Liyan Chen, **e-mail**: lychen@ybu.edu.cn,

Zhenhua Lin, **e-mail**: zhlin720@ybu.edu.cn

Supplemental figures legends:

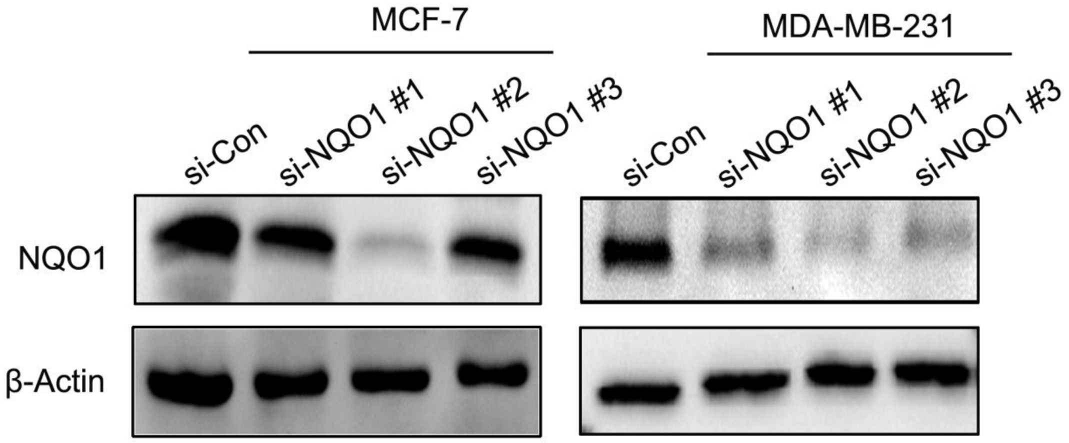

Supplementary Figure 1: NQO1 was knocked down by siRNA in breast cancer cells. NQO1 protein expression was inhibited in si-NQO1-transfected MCF-7 and MDA-MB-231 cells, as demonstrated by western blot.

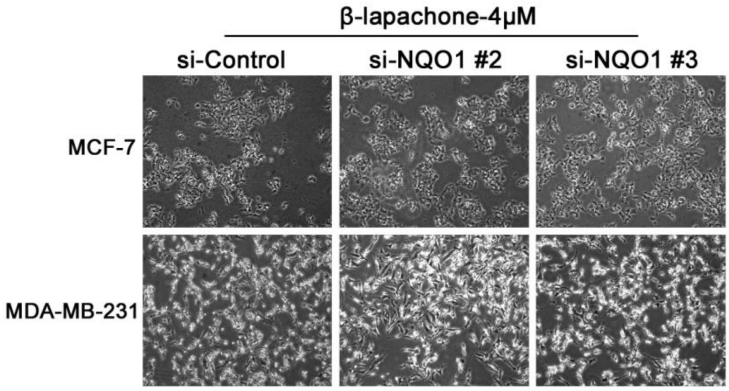

Supplementary Figure 2: β-lapachone suppressed cell viability of MCF-7 and MDA-MB-231 cells in NQO1-dependent manner. NQO1 knockdown significantly protected breast cancer cells from β-lapachone-induced cell death in MCF-7 and MDA-MB-231 cells

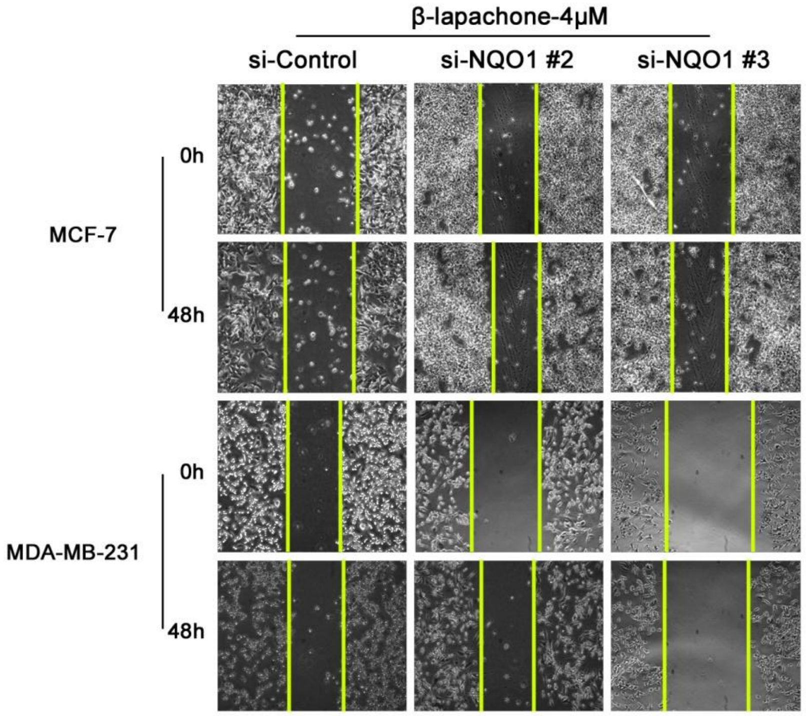

Supplementary Figure 3: β-lapachone suppressed the ability of cell migration on MCF-7 and MDA-MB-231 cells in NQO1-dependent manner. The inhibition on the migration of MCF-7 and MDA-MB-231 cells by β-lapachone were significantly reduced after NQO1 knocked down.

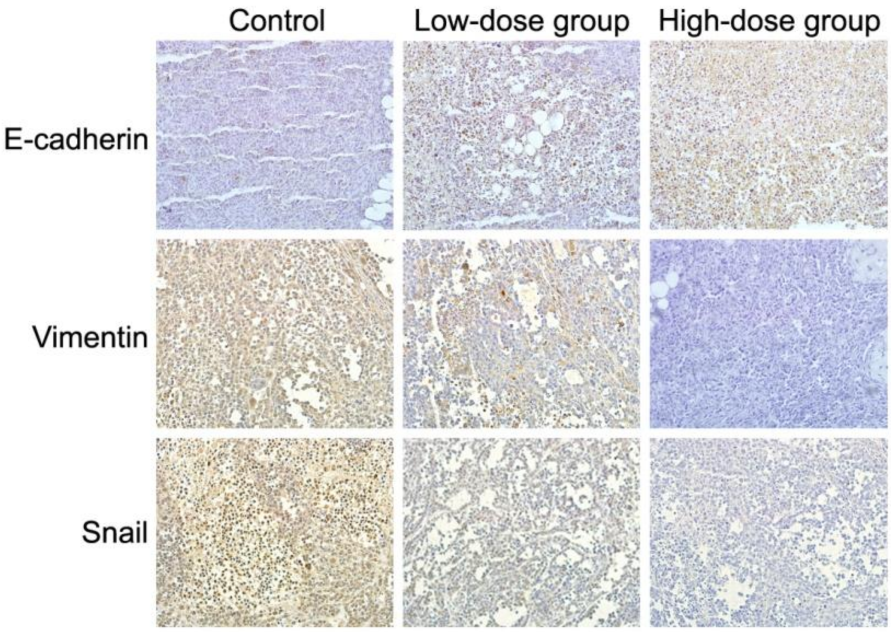

Supplementary Figure 4: β-lapachone effectively attenuate the EMT process of breast cancer cells in a mouse xenograft model. The expression levels of E-cadherin, Vimentin and snail in the tumour tissues were assayed by IHC staining (200×).

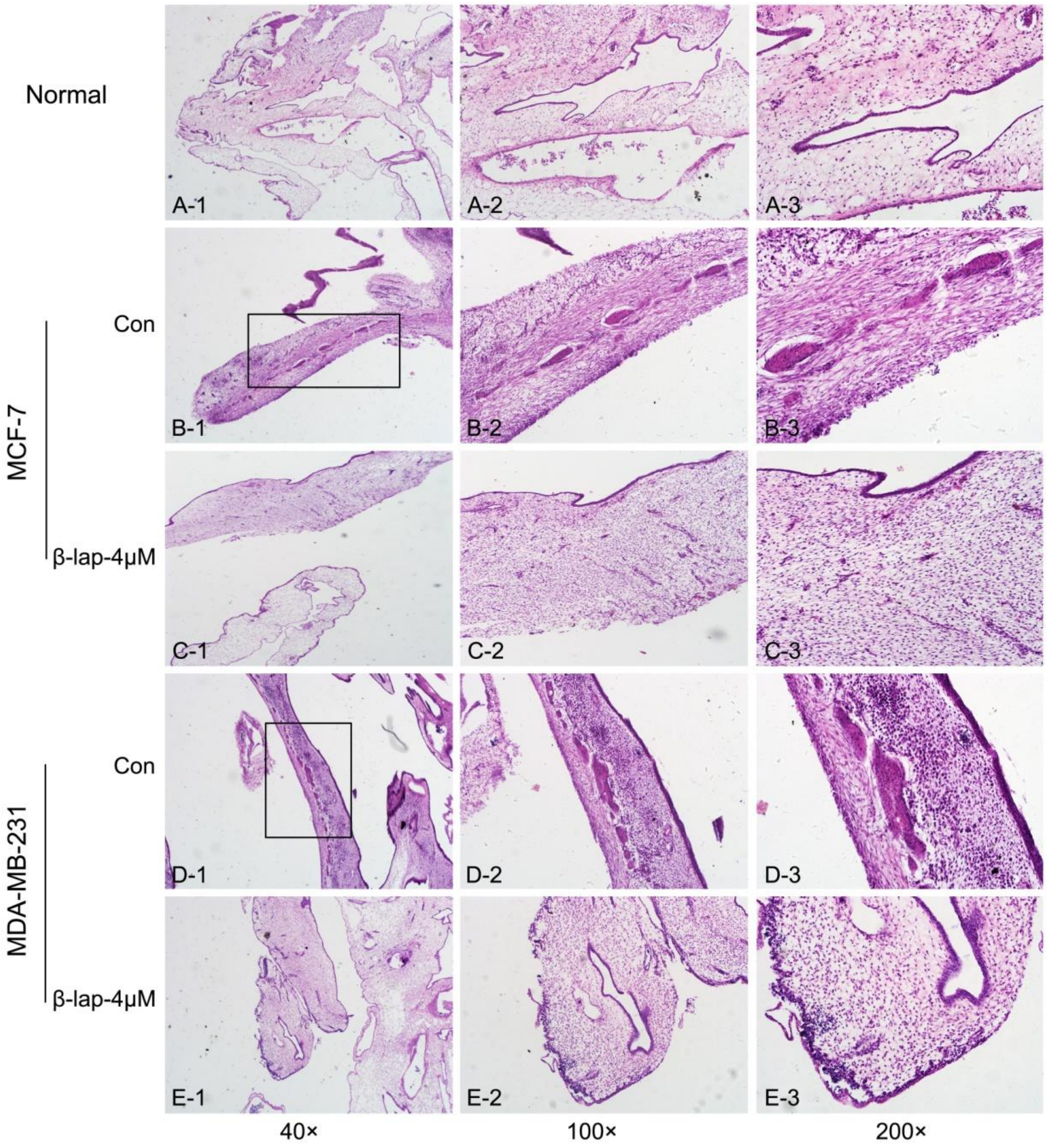

Supplementary Figure 5: Invasion of breast cancer cells in the chick chorioallantoic membrane (CAM) by H&E staining. (A1-A3) Normal structure of CAM layers. (B1-B3, C1-C3) MCF-7 cells which treated with β-lapachone showed less invasion into the CAM than control cells. (D1-D3, E1-E3) MDA-MB-231 cells which treated with β-lapachone showed less invasion into the CAM than control cells.
